# Bone Marrow Edema Syndrome as an Emerging Clinical Condition: Investigating the Link with Bone Status—A Single-Center Study

**DOI:** 10.3390/jcm15155973

**Published:** 2026-07-31

**Authors:** Carla Caffarelli, Antonella Al Refaie, Guido Cavati, Alessandro Versienti, Sara Gonnelli, Luigi Gennari, Bruno Frediani, Stefano Gonnelli, Caterina Mondillo

**Affiliations:** 1 Department of Medicine, Surgery and Neuroscience, University of Siena, 53100 Siena, Italy; antonellaalrefaie@gmail.com (A.A.R.); guido.cavati@student.unisi.it (G.C.); versientialessandro@gmail.com (A.V.); saragonnelli@libero.it (S.G.); luigi.gennari@unisi.it (L.G.); gonnelli@unisi.it (S.G.); caterinamondillo28@gmail.com (C.M.); 2 Division of Internal Medicine I, San Giuseppe Hospital, 50053 Empoli, Italy; 3 Department of Geriatrics, University Hospital of Nice, 06000 Nice, France

**Keywords:** bone marrow edema syndrome (BMEs), bone mineral density (BMD), trabecular bone score (TBS), complex regional pain syndrome type 1 (CRPS-1), osteoporosis, vitamin D, bone turnover markers

## Abstract

**Background:** Bone Marrow Edema Syndrome (BMEs) encompasses primary clinical conditions characterized by pain and high-intensity signals on fluid-sensitive magnetic resonance imaging at the subchondral bone level. This study aimed to evaluate bone mineral density (BMD) and turnover markers in BMEs patients compared to a healthy control group. **Methods:** We selected from the records 165 patients—comprising Complex Regional Pain Syndrome type 1 (CRPS-1, *n* = 61), hip BMEs (*n* = 42), and knee BMEs (*n* = 62)—alongside 150 age- and sex-matched healthy controls. Assessments included bone turnover markers, vitamin D levels, and BMD at the lumbar spine and femur by Dual-Energy X-ray Absorptiometry. Trabecular Bone Score (TBS) was calculated to estimate bone microarchitecture. **Results:** BMEs patients demonstrated significantly lower BMD across all skeletal sites and reduced TBS values compared to controls. Osteoporosis prevalence was significantly higher in all BMEs subgroups, with CRPS-1 and hip BMES patients showing higher rates than those with knee BMEs. No significant differences were observed in vitamin D or bone turnover markers between groups. **Conclusions:** Patients with CRPS-1 and BMEs of the hip or knee exhibit a higher prevalence of osteoporosis and compromised bone microarchitecture. These findings highlight a significant association between BME-related conditions and altered BMD/TBS parameters, suggesting a potential shared pathophysiological link.

## 1. Introduction

Bone marrow edema (BME) is a radiological descriptor for areas characterized by low signal intensity on T1-weighted magnetic resonance imaging (MRI) and high signal intensity on T2-weighted or short tau inversion recovery (STIR) sequences [1]. The term was coined in 1988 by Wilson et al. to describe increased water content in the bone marrow of patients presenting with significant hip or knee pain [2]. In 2004, Hofmann et al. introduced the broader term “bone marrow edema syndrome” (BMEs) as a diagnosis of exclusion for clinical conditions involving pain and increased interstitial fluid in the bone marrow without an identifiable underlying cause [3].

The integration of MRI into clinical practice has led to various overlapping terms, such as “transient osteoporosis,” “transient demineralization,” and “regional migratory osteoporosis.” A recent scoping review by Gravle on this nomenclature noted that while many publications use osteoporosis-related terminology, few studies actually measured bone mineral density (BMD). Consequently, it was suggested that the overarching terminology should focus on bone marrow edema rather than osteoporosis [4].

While the pathophysiology of BMEs remains a subject of debate, previous research has identified minor trauma as a potential risk factor [5]. However, emerging evidence suggests that bone metabolic damage may be the primary driver of BMEs, influencing its clinical features, diagnosis, and treatment [6]. Recently, Molfetta et al. argued that Complex Regional Pain Syndrome type 1 (CRPS-1)—despite its distinct clinical presentation—should be categorized within the BMEs spectrum due to its shared imaging characteristics, pathophysiology, and positive therapeutic response to bisphosphonates [6]. A hallmark of BMEs is the preservation of the articular surface alongside varying degrees of pain [4].

CRPS-1 is a rare clinical condition that typically affects a single limb. It is more prevalent in females, with a reported ratio between 2:1 and 4:1. The syndrome is characterized by intense pain disproportionate to the inciting event, often accompanied by vasomotor, sudomotor, sensorimotor, and trophic alterations [7,8]. Historically, bone involvement was central to the disease; Paul Sudeck identified “patchy osteoporosis” as a pivotal diagnostic radiological feature [9]. Despite the known roles of fractures, sprains, and skeletal microtrauma in triggering CRPS-1, recent decades have seen a decline in focus on bone involvement. Indeed, the current Budapest criteria from the International Association for the Study of Pain (IASP) rely exclusively on clinical signs and symptoms, omitting imaging [10]. Therefore, although increasing evidence suggests that CRPS-1 and BMEs may share common mechanisms related to altered bone metabolism and that both conditions may respond to antiresorptive therapy, their relationship remains controversial. Bone marrow edema is the hallmark MRI finding of BMEs, whereas in CRPS-1 it is neither a diagnostic requirement nor a consistent imaging feature. MRI evidence of bone marrow edema is observed mainly during the early “warm” stage of CRPS-1 and is absent in a substantial proportion of patients. Moreover, the prominent autonomic, sensory, and trophic disturbances that characterize CRPS-1 distinguish it clinically from BMEs. Consequently, CRPS-1 may be considered to overlap with BMES in selected cases or to share part of the same pathophysiological spectrum, but the two conditions cannot currently be regarded as fully equivalent [11].

The femur is the skeletal site most frequently affected by BMEs, involving both proximal and distal regions without a clear etiology. Transient Osteoporosis of the Hip (TOH) is the most common manifestation of BMEs in the hip [12]. TOH is an idiopathic, self-limiting condition characterized by hip pain and limping, predominantly affecting middle-aged men and women during the third trimester of pregnancy or the early postpartum period. Pain typically intensifies with weight-bearing and at night, subsiding with rest. MRI is the gold standard for early detection, identifying TOH within days of symptom onset [6,13,14]. Notably, a significant portion of TOH patients exhibit low BMD, although densitometry is not routinely performed [12,13,15].

Primary BMEs of the knee presents either as transient osteoporosis of the knee or, more frequently, as Regional Migratory Osteoporosis (RMO). RMO is characterized by arthralgia in a single joint that subsequently “migrates” to another location, usually more distal, after several months [16,17]. Pathogenetically and radiologically, RMO and TOH are identical. Furthermore, “transient BMES of the knee” is clinically and radiologically indistinguishable from transient osteoporosis on MRI; the former is often considered the clinical precursor to detectable osteopenia [16,18]. Recent studies also suggest that TOH and RMO are unlikely to progress directly to osteonecrosis [1,19,20,21].

This single-center observational retrospective study aimed to evaluate axial BMD (via DXA), the Trabecular Bone Score (TBS), and bone turnover markers in patients diagnosed with BMEs (including CRPS-1, hip BMEs, and knee BMEs) at the time of diagnosis and prior to the initiation of pharmacological therapy.

## 2. Materials and Methods

### 2.1. Study Design and Patient Population

**Figure 1:**
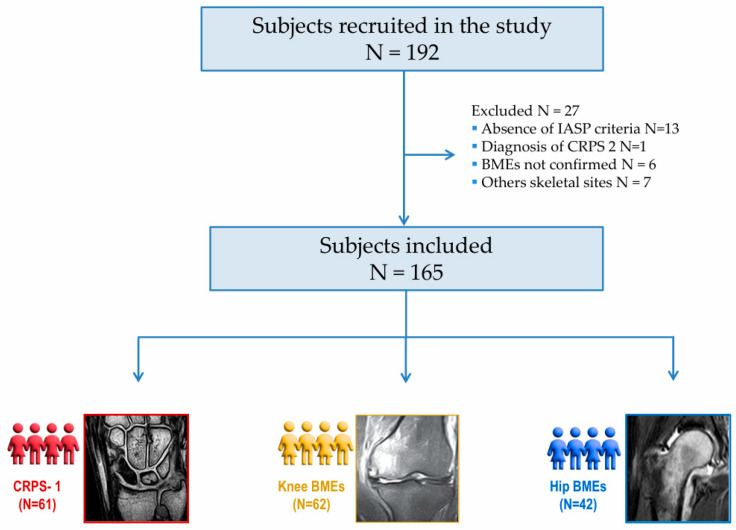
Flow chart showing the distribution of subjects with BMEs.

Approved by the Ethics Committee on 13 December 2021, this study includes patients selected from the outpatient clinic of the Internal Medicine Unit at the University Hospital of Siena (Siena, Italy) as part of a broader ambispective design. The design comprised a retrospective phase (January 2018 to December 2021) and a prospective phase (December 2021 to December 2025). To be eligible for enrollment, patients were required to present an MRI-confirmed diagnosis of either CRPS-1 or BMEs of the hip or knee. The radiological criteria for these conditions were defined by the presence of abnormal signal patterns, specifically well-defined areas of hypointensity on T1-weighted sequences and hyperintensity on T2-weighted or STIR sequences. Furthermore, participants had to be between 18 and 70 years of age and report significant spontaneous pain in the affected limb, quantified as ≥50 mm on a 100 mm Visual Analogue Scale during the week preceding the assessment. Several exclusion criteria were applied to ensure the homogeneity of the cohort. Patients with a disease duration exceeding six months, a history of severe chronic illnesses, or concurrent chronic pain syndromes that might interfere with pain ratings were excluded. Regarding pharmacological history, the study excluded individuals previously treated with bisphosphonates or other anti-osteoporosis medications—with the exception of calcium and vitamin D supplements—as well as those undergoing therapies known to influence bone metabolism, such as glucocorticoids, glitazones, or anticonvulsants. For cases of suspected CRPS-1, patients were excluded if they did not meet the IASP “Budapest” diagnostic criteria or if they exhibited major nerve damage suggestive of CRPS-2. Out of an initial pool of 192 evaluated patients, 27 were excluded for the following reasons: 13 failed to satisfy the Budapest criteria, 7 showed BME involvement in skeletal sites other than the hip or knee, and one presented with nerve damage indicative of CRPS-2, 6 were diagnosed with secondary BMEs. The final study population consisted of 165 patients, subdivided into three groups: CRPS-1 (*n* = 61), hip BMEs (*n* = 42), and knee BMEs (*n* = 62), as illustrated in the participant flow chart (Figure 1). To maintain diagnostic precision, all MRI examinations were reviewed by an experienced radiologist prior to recruitment to specifically rule out bone edema secondary to osteoarthritis. A control group of 150 healthy individuals, matched for age and sex, was established using hospital personnel and participants from a local epidemiological study. Female controls were also matched for menopausal status. Comprehensive clinical assessments were performed for all participants, including the measurement of vital signs and anthropometric data. Body Mass Index (BMI) was calculated as the ratio of weight in kilograms to the square of height in meters (kg/m^2^). Detailed personal and family medical histories were also recorded. The study was conducted in compliance with the Declaration of Helsinki and received approval from the Ethics Committee of Siena University Hospital (ID-21211). All participants provided informed written consent, and all data were strictly anonymized before statistical analysis.

### 2.2. Biochemical and Bone Turnover Assessments

Following an overnight fast of at least 12 h, venous blood samples were collected from all participants to evaluate a comprehensive metabolic and bone turnover profile. The laboratory assessment included the measurement of glycemia, creatinine, calcium, phosphate, albumin, total alkaline phosphatase, and parathyroid hormone (PTH). Specific markers of bone metabolism were also quantified, including 25-hydroxyvitamin D (25OHD), bone-specific alkaline phosphatase (B-ALP), and C-terminal telopeptide of type 1 collagen (β-CTX). Regarding the analytical methods, serum β-CTX was determined via an ELISA kit (Immunotopics INT, San Clemente, CA, USA), which demonstrated an intra-assay precision of 2.5% and an inter-assay precision of 3.5%. Serum B-ALP levels were measured using a chemiluminescence immunoassay (LIAISON BAP Ostase, DiaSorin Inc., Stillwater, MN, USA), with intra- and inter-assay coefficients of variation (CV) of 4.2% and 7.9%, respectively. Furthermore, serum PTH was assessed by immunoradiometric assay (Total Intact PTH, Antibodies Lab. Inc., Santee, CA, USA), showing intra- and inter-assay CVs of 3.6% and 4.9%. Finally, serum 25OHD concentrations were determined by a chemiluminescence immunoassay (LIAISON 25OHD Total Assay, DiaSorin Inc., Stillwater, MN, USA), for which our institution recorded intra- and inter-assay CVs of 6.8% and 9.2%, respectively.

### 2.3. Bone Mineral Density Assessment

Bone Mineral Density (BMD) was assessed in all subjects at the lumbar spine (LS-BMD), femoral neck (FN-BMD), and total hip (TH-BMD) using a dual-energy X-ray absorptiometry (DXA) system (Discovery W, Hologic, Waltham, MA, USA). All scans were conducted in accordance with standardized clinical protocols. Notably, femoral BMD measurements were obtained from the limb contralateral to the site affected by BMEs to ensure accuracy. The diagnosis of osteoporosis and osteopenia followed the World Health Organization (WHO) criteria, where a T-score ≤ −2.5 was defined as osteoporosis and a T-score between −1.0 and −2.5 as osteopenia; these scores were calculated using sex-matched Italian reference databases.

### 2.4. Trabecular Bone Score

To further evaluate bone microarchitecture, the Trabecular Bone Score (TBS) was determined from the anteroposterior lumbar spine scans using an automated, operator-independent process with TBS iNsight software (Version 2.1, Medimaps SA, Bordeaux, France). TBS measurements were calibrated using a standard phantom (17 cm thickness, 25% fat mass equivalent) and adjusted for a BMI of 21.78. In our clinical setting, the short-term precision for TBS, expressed as the coefficient of variation (CV), was 1.5%.

### 2.5. Statistical Analysis

Continuous variables are expressed as mean ± standard deviation (SD). The normality of the data distribution was verified using the Kolmogorov–Smirnov test to determine the appropriate subsequent statistical approach. Differences in clinical characteristics and baseline parameters between the study groups were analyzed using Student’s *t*-test for normally distributed data or the Mann–Whitney U-test for non-parametric variables. Accordingly, differences among the three independent groups were analyzed using a One-Way ANOVA followed by Tukey’s post-hoc test for normally distributed parameters, or the non-parametric Kruskal–Wallis test followed by Dunn’s post-hoc test for non-normally distributed data. For the analysis of categorical data, the Chi-square test or Fisher’s exact test was applied as appropriate. Correlation analyses between the various parameters were conducted using Pearson’s or Spearman’s coefficients, depending on the distribution, or through partial correlation analysis where required. All statistical procedures were carried out using the SPSS software package for Windows, version 16.0 (SPSS Inc., Chicago, IL, USA), with significance levels maintained according to standard protocols. Moreover, given the number of comparisons across different skeletal sites, biochemical markers, and age subgroups, no formal multiplicity correction was pre-specified. Therefore, all subgroup evaluations and partial correlation analyses were considered exploratory in nature, aimed at hypothesis generation. A nominal *p* < 0.05, was used to indicate statistical significance, with borderline findings interpreted with caution.

## 3. Results

Clinical and densitometric characteristics for both the BMEs cohort and the age- and sex-matched healthy controls are detailed in Table 1. The analysis confirmed that the cohorts were well-balanced with respect to anthropometric variables, including age and BMI, thereby minimizing potential confounding effects. Conversely, a comparative analysis of bone health parameters revealed a marked reduction in BMD across all examined skeletal sites in the BMEs group (*p* < 0.01). This systemic reduction in bone mass was accompanied by significantly lower TBS values compared to the control group, indicating a concurrent compromise of the bone microarchitectural integrity in patients with BMEs.

The clinical characteristics and biochemical parameters of patients grouped by the localization of BMEs are shown in Table 2. Patients with knee BME exhibited a significantly higher BMI than those in the CRPS-I and hip BME groups. Time from diagnosis averaged 4.0 ± 4.2, 4.8 ± 3.8 and 3.3 ± 4.0 weeks for CRPS-I, knee BME, and hip BME, respectively. Regarding biochemical markers, B-ALP and β-CTX values were significantly higher in the knee BME cohort (*p* < 0.05); however, other parameters—including calcium, phosphate, total ALP, vitamin D, and PTH—showed no significant variation across the three groups.

Table 3 displays the partial correlations, adjusted for age and BMI, between bone turnover markers (B-ALP and β-CTX) and BMD at all skeletal sites, categorized by BME localization. In patients with knee BMEs, both B-ALP and β-CTX showed significant correlations with BMD across all measured sites. Notably, in the Hip BME and CRPS-I subgroups, B-ALP showed no significant correlation with BMD values across all measured sites, whereas β-CTX showed correlations with BMD at all skeletal sites in the Knee BME subgroup and only with LS-BMD in the CRPS-I subgroup.

**Figure 2:**
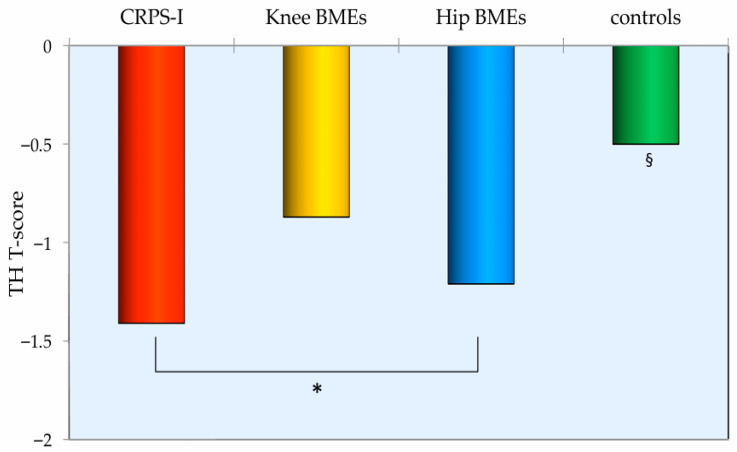
Values of BMD expressed as T-score at total hip (TH) in patients according to with BMEs localization and in healthy controls (* *p* < 0.05 ANOVA between BMEs localization; ^§^ *p* < 0.01 between BMEs vs. controls).

Figure 2 illustrates the mean TH-BMD, expressed as T-scores, for both BMEs patients and healthy controls. BMD T-scores were significantly lower in the BMEs group compared to the control group (*p* < 0.05). Furthermore, when stratified by BMEs localization, patients with CRPS-I exhibited the lowest total hip T-scores.

**Figure 3:**
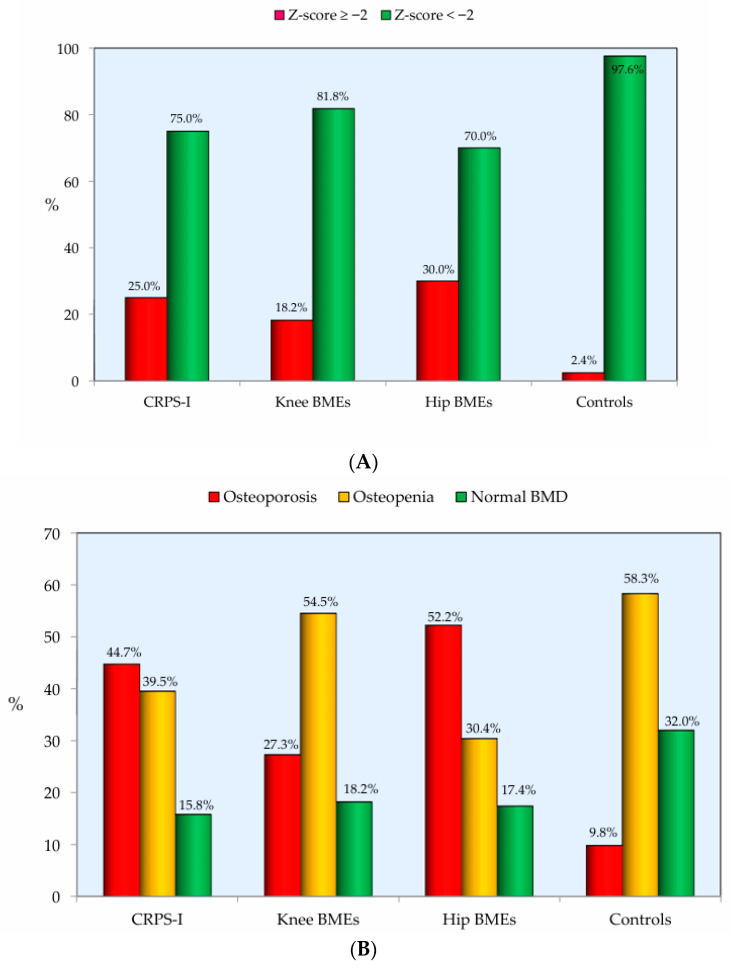
(**A**) Percentage of subjects with a Z-score ≥ −2 and a Z-score < −2 in patients and healthy controls aged under 50 years. (**B**) Percentage of “osteoporosis”, “osteopenic” or “normal BMD” in patients and healthy controls aged 50 years and older.

Figure 3A,B illustrate the distribution of BME patients and healthy controls stratified by age, according to WHO criteria. In the cohort aged under 50 years (Figure 3A), evaluated using Z-scores, all three BME subgroups exhibited a significantly higher prevalence of diminished bone density compared to the healthy control group. Notably, the proportion of patients falling into the osteoporotic range was more pronounced in the CRPS-I and hip BME cohorts than in the knee BME group. A consistent trend was observed in the cohort aged 50 years and older (Figure 3B). The prevalence of osteoporosis remained significantly elevated across all BME conditions relative to controls, with CRPS-I and hip BME patients continuing to demonstrate the most severe reduction in bone mineral density.

**Figure 4:**
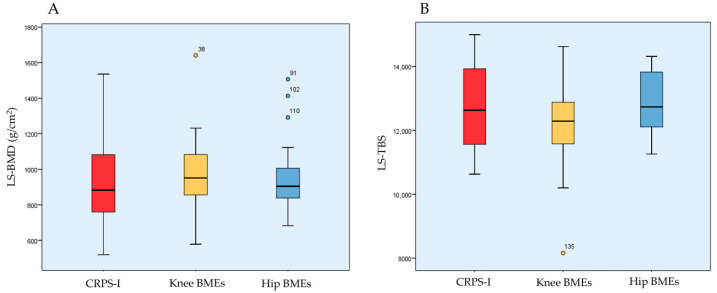

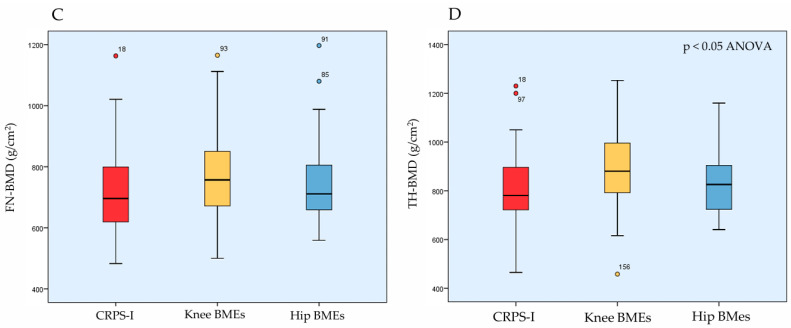
BMD-LS (**A**), TBS (**B**), BMD-FN (**C**) and BMD-TH (**D**) in patients with CPRS-I, knee BMEs and hip BMEs.

Figure 4 illustrates the BMD and TBS values stratified by BME localization. Specifically, Figure 4A,B represent the BMD-LS and TBS across the BMEs subgroups, stratified by disease location. While patients with knee BME exhibited higher BMD-LS values than those with CRPS-I or hip BME, this trend did not reach statistical significance. Similarly, no significant intergroup differences were observed in TBS values. In Figure 4C,D BMD values for the BMD-FN and BMD-TH are shown. Although both BMD-FN and BMD-TH were lower in the CRPS-I and hip BME cohorts compared to the knee BME group, the difference was statistically significant only for the total hip (*p* < 0.05).

## 4. Discussion

The primary finding of this study is that patients with bone marrow edema syndromes, particularly those presenting with CRPS-I and hip BME, exhibit significantly lower BMD compared to age- and sex-matched healthy controls. Over the last two decades, multiple studies have utilized DXA to document reduced BMD in CRPS-I patients, noting bone loss not only in the affected limb but also in the contralateral limb and at the lumbar spine [22,23]. Furthermore, a high prevalence of osteopenia and osteoporosis has been reported in this population [22,23]. Notably, a large-scale Dutch population study by de Mos et al. identified menopause and osteoporosis as critical risk factors for the development of CRPS-I [23].

To the best of our knowledge, this is the first study to demonstrate that the Trabecular Bone Score (TBS)—a densitometric index reflecting bone microarchitecture and quality—is significantly lower in patients with BMEs compared to healthy controls. These findings complement the work of Oehler et al. [24], who utilized high-resolution peripheral quantitative computed tomography (HR-pQCT) to identify profound cortical and trabecular microstructural alterations in CRPS-I. Together, these data provide novel insights into the specific morphological and pathophysiological mechanisms driving bone loss in this condition. The potential link between CRPS-I and bone quantity/quality is further corroborated by documented cases of Sudeck’s atrophy in patients with osteogenesis imperfecta [25]. Despite these clinical observations and the established role of fractures or bone trauma as inciting events, research and taxonomic classifications have historically focused on sympathetic hyperactivation as the primary pathogenic mechanism. However, more recent perspectives have shifted this focus; notably, Varenna and Crotti [26] identified bone involvement as a hallmark of CRPS-I, proposing a pathophysiological model centered on bone-derived signals. In this framework, direct bone injury triggers a localized release of proinflammatory mediators—including TNF, IL-1, IL-6, substance P, and calcitonin gene-related peptide—leading to altered capillary permeability, subsequent edema, hypoxia, and tissue acidosis [13,26]. Another noteworthy finding of our study is that bone turnover markers (B-ALP and β-CTX) remained within physiological limits, despite the majority of our cohort being postmenopausal women. These data align with recent evidence challenging the paradigm of accelerated bone turnover and osteoclast hyperactivity in the early stages of CRPS-I [13,27]. Instead, it is increasingly suggested that the rapid decline in BMD observed during these phases may stem from the chemical dissolution of hydroxyapatite, driven by localized acidosis and a significant drop in microenvironmental pH [13,27]. In the present study, we observed neither reduced vitamin D levels nor elevated PTH concentrations, whether considering the entire cohort or analyzing the three subgroups (CRPS-I, hip BMEs, and knee BMEs) independently. These results appear to contrast with several previous reports [28,29]. Specifically, a recent scoping review indicated that over 60% of studies involving patients with BME syndromes reported vitamin D levels consistent with deficiency or insufficiency [30]. This discrepancy may be attributed to regional clinical practices, as the majority of postmenopausal women in our geographical area undergo regular vitamin D supplementation, potentially masking the deficiencies observed in other populations.

In the present study, the prevalence of osteoporosis among patients with hip BMEs was nearly identical to that observed in the CRPS-I group, and markedly higher than in patients with knee BMEs. This finding is particularly noteworthy, as it suggests distinct physiological characteristics that may differentiate hip BME from knee BME. Our results align with the majority of studies on primary hip BME, which identify low bone mass—either osteopenia or osteoporosis—as the most significant risk factor for the onset of the condition [15,31]. Indeed, a large case series from the Mayo Clinic [17] and a recent narrative review [16] found that over 50% of patients with transient osteoporosis of the hip (TOH) exhibited DXA-derived BMD values consistent with osteoporosis or osteopenia. Similarly, in a cohort of 31 patients with TOH, Asadipooya et al. [32] reported that 15 individuals were osteopenic and 15 were osteoporotic based on lumbar spine densitometry. Furthermore, a predisposition to BME syndromes has been hypothesized in patients with underlying bone fragility, such as those with osteogenesis imperfecta [15,25]. However, it is important to emphasize that in most previous studies, BMD was assessed only in a small subgroup of patients, and it remains unclear whether the affected (and potentially edematous) limb was excluded from the densitometric analysis to avoid confounding results.

Our analysis revealed that patients with knee BMEs exhibit significantly higher levels of bone turnover markers compared to those with CRPS-I or hip BMEs. Nevertheless, it is crucial to note that both B-ALP and β-CTX concentrations remained within the physiological reference range across all groups. It has been hypothesized that in both hip and knee BME syndromes—similar to CRPS-I—the rapid reduction in bone mass within the proximal or distal femur does not stem from osteoclastic hyperactivity. Instead, this phenomenon may be attributed to the chemical dissolution of hydroxyapatite crystals, triggered by localized acidosis and a reduction in microenvironmental pH [13]. Within this framework, the observed increase in B-ALP levels may reflect a compensatory enhancement of osteoblastic activity, which could also account for the elevated osteoprotegerin levels reported in similar clinical contexts [33].

Our study has certain limitations that warrant consideration. Primarily, its retrospective design precludes a definitive analysis of the causal relationships between specific risk factors, symptom intensity or duration, and therapeutic outcomes. Additionally, the diagnosis of primary BME involving the hip and knee remains a diagnosis of exclusion, which inherently carries the risk of including patients with confounding underlying conditions. Despite these limitations, the study possesses significant strengths. First, it is a single-center study involving a large and well-characterized cohort, all of whom were evaluated using uniform and predefined diagnostic criteria. Second, to the best of our knowledge, this is one of the very few studies to have systematically assessed BMD via DXA in all patients with BME prior to the initiation of any pharmacological therapy. Finally, this is the first study to evaluate the Trabecular Bone Score in this clinical population, providing novel insights into the microarchitectural quality of bone in BMEs.

## 5. Conclusions

In conclusion, this study demonstrates a significant cross-sectional association between Bone Marrow Edema (BME)-related conditions—particularly CRPS-I and hip involvement—and a higher prevalence of altered bone density, alongside a meaningful reduction in TBS compared to age- and sex-matched healthy controls. These convergent observations, together with shared MRI features and a comparable clinical response to bisphosphonates, suggest a potential common pathway involving bone tissue vulnerability. Although our cross-sectional design cannot establish direct causal mechanisms or temporal direction, these cumulative findings provide a clinically and biologically plausible rationale to hypothesize that these disorders may be grouped under the unifying spectrum of ‘Bone Marrow Edema Syndromes’ warranting further longitudinal validation.

## Figures and Tables

**Table 1:** Baseline clinical and densitometric characteristics of the BMEs cohort and healthy controls.

	BMEs (*n* = 165)	Controls (*n* = 150)
Sex (F/M)	96/69	88/62
Age (yrs)	58.1 ± 11.9	55.7 ± 12.1
BMI (Kg/m^2^)	25.7 ± 3.9	26.3 ± 4.6
LS-BMD (g/cm^2^)	0.958 ± 0.196	1.098 ± 0.145 *
LS-T-score	−1.37 ± 1.71	−0.68 ± 1.17 *
LS-Z-score	−0.30 ± 1.62	0.22 ± 1.13 *
TBS	1.251 ± 0.149	1.392 ± 0.087 *
FN-BMD (g/cm^2^)	0.749 ± 0.151	0.869 ± 0.111 *
FN-T-score	−1.48 ± 1.02	−1.03 ± 0.89 *
FN-Z-score	−0.37 ± 0.97	0.01 ± 0.77 *
TH-BMD (g/cm^2^)	0.861 ± 0.139	0.951 ± 0.118 *
TH-T-score	−1.11 ± 0.99	−0.62 ± 0.91 *
TH-Z-score	−0.32 ± 0.99	0.12 ± 0.80 *

* *p* < 0.01 BMEs vs. Controls.

**Table 2:** Clinical and biochemical characteristics of the patients with BMEs.

	CRPS-I (*n* = 61)	Knee BMEs (*n* = 62)	Hip BMEs (*n* = 42)
Sex (F/M)	41/20	31/31	24/18
Age (yrs)	58.7 ± 9.6	59.3 ± 12.1	58.5 ± 13.1
BMI (Kg/m^2^)	25.3 ± 4.2	27.4 ± 4.5	25.1 ± 3.9 *
Calcium (mg/dL)	9.4 ± 0.4	9.4 ± 0.5	9.4 ± 0.3
Phosphate (mg/dL)	3.3 ± 0.5	3.4 ± 0.4	3.3 ± 0.6
Creatinine (mg/dL)	0.7 ± 0.1	0.8 ± 0.2	0.9 ± 0.2 *
Alkaline phosphatase (U/L)	80.1 ± 38.3	78.8 ± 22.4	81.5 ± 32.5
25OHD (ng/mL)	25.8 ± 13.9	26.8 ± 14.1	27.1 ± 14.9
PTH (pg/mL)	31.8 ± 15.8	32.7 ± 17.8	32.4 ± 18.7
B-ALP (µg/L)	13.2 ± 6.1	15.4 ± 13.9	13.7 ± 6.9 *
β-CTX (ng/L)	0.477 ± 0.405	0.526 ± 0.243	0.449 ± 0.248 *

* *p* < 0.05 for pairwise comparisons by Kruskal–Wallis test between BME groups (knee BME vs. CRPS-I and hip BME).

**Table 3:** Age and BMI-adjusted partial correlation between β-CTX and B-ALP and BMD at all skeletal sites according to with BMEs localization.

	LS-BMD (g/cm^2^)	FN-BMD (g/cm^2^)	TH-BMD (g/cm^2^)
**CRPS-I**			
B-ALP (µg/L)	−0.27	−0.19	−0.18
β-CTX (ng/L)	−0.57 **	−0.24	−0.23
**Knee BMEs**			
B-ALP (µg/L)	−0.52 **	−0.54 **	−0.56 **
β-CTX (ng/L)	−0.55 **	−0.62 **	−0.74 **
**Hip BMEs**			
B-ALP (µg/L)	0.29	0.31	0.28
β-CTX (ng/L)	−0.63 **	−0.48 **	−0.47 *

(* *p* < 0.05, ** *p* < 0.01 indicate statistically significant correlations).

## Data Availability

The data presented in this study are available on request from the corresponding author.

## Abbreviations

The following abbreviations are used in this manuscript:

BMEs	Bone marrow edema syndrome
CRPS-1	Complex Regional Pain Syndrome type 1
BMD	Bone mineral density
MRI	Magnetic resonance imaging
STIR	Short tau inversion recovery
IASP	Association for the Study of Pain
TOH	Transient Osteoporosis of the Hip
RMO	Regional Migratory Osteoporosis
DXA	Dual-Energy X-ray Absorptiometry
BMI	Body mass index
PTH	Parathyroid hormone
25OHD	25-hydroxyvitamin D
B-ALP	Bone-specific alkaline phosphatase
Β-CTX	C-terminal telopeptide of type 1 collagen
LS	Lumbar spine
FN	Femoral neck
TH	Total hip

## References

[1] Korompilias A.V., Karantanas A.H., Lykissas M.G., Beris A.E. (2009). Bone marrow edema syndrome. Skelet. Radiol..

[2] Wilson A.J., Murphy W.A., Hardy D.C., Totty W.G. (1988). Transient osteoporosis: Transient bone marrow edema?. Radiology.

[3] Hofmann S., Kramer J., Vakil-Adli A., Aigner N., Breitenseher M. (2004). Painful bone marrow edema of the knee: Differential diagnosis and therapeutic concepts. Orthop. Clin. N. Am..

[4] Grøvle L., Julsrud Haugen A., Johansen M., Hasvik E. (2024). The terminologies of transient, migratory, or localized osteoporosis, and bone marrow edema syndrome: A scoping review. Osteoporos. Int..

[5] Hasvik E., Haugen A.J., Grøvle L. (2025). Clinical characteristics of patients with bone marrow edema syndrome, transient osteoporosis or migratory osteoporosis: A scoping review. Bone.

[6] Hasvik E., Haugen A.J., Grøvle L. (2026). A bibliometric analysis of research on bone marrow edema syndrome, transient osteoporosis of the hip, regional migratory osteoporosis, and related terms. Medicine.

[7] Manara M., Varenna M. (2014). A clinical overview of bone marrow edema. Reumatismo.

[8] Molfetta L., Florian A., Saviola G., Frediani B. (2022). Bone Marrow Edema: Pathogenetic features. Clin. Ter..

[9] Marinus J., Moseley G.L., Birklein F., Baron R., Maihofner C., Kingery W.S., van Hilten J.J. (2011). Clinical features and pathophysiology of complex regional pain syndrome. Lancet Neurol..

[10] Ferraro M.C., E O’cOnnell N., Sommer C., Goebel A., Bultitude J.H., Cashin A.G., Moseley G.L., McAuley J.H. (2024). Complex regional pain syndrome: Advances in epidemiology, pathophysiology, diagnosis, and treatment. Lancet Neurol..

[11] Sudeck P. (1900). Über die acute entzündliche knochenatrophie. Arch. Klin. Chir..

[12] Harden R.N., Oaklander A.L., Burton A.W., Perez R.R.S.G.M., Richardson K., Swan M., Barthel J., Costa B., Graciosa J.R., Bruehl S. (2013). Complex regional pain syndrome: Practical diagnostic and treatment guidelines, 4th edition. Pain Med..

[13] Kollmann G., Wertli M.M., Dudli S., Distler O., Brunner F. (2023). The role of the bone in complex regional pain syndrome 1—A systematic review. Eur. J. Pain.

[14] Patel S. (2014). Primary bone marrow oedema syndromes. Rheumatology.

[15] Trevisan C., Klumpp R., Compagnoni R. (2016). Risk factors in transient osteoporosis: A retrospective study on 23 cases. Clin. Rheumatol..

[16] Di Martino A., Brunello M., Villari E., Cataldi P., D’Agostino C., Faldini C. (2023). Bone marrow edema of the hip: A narrative review. Arch. Orthop. Trauma Surg..

[17] Kotwal A., Hurtado M.D., Sfeir J.G., Wermers R.A. (2019). Transient osteoporosis: Clinical spectrum in adults and associated risk factors. Endocr. Pract..

[18] Roemer F., Frobell R., Hunter D., Crema M.D., Fischer W., Bohndorf K., Guermazi A. (2009). MRI-detected subchondral bone marrow signal alterations of the knee joint: Terminology, imaging appearance, relevance and radiological differential diagnosis. Osteoarthr. Cartil..

[19] Komnos G.A., Paridis D.I., Banios K.T., Karachalios T.S., Dailiana Z.H., Luceri F., Randelli P.S. (2023). Regional migratory osteoporosis of the knee: A literature overview. Musculoskelet. Surg..

[20] Geith T., Stellwag A.C., Müller P.E., Reiser M., Baur-Melnyk A. (2020). Is bone marrow edema syndrome a precursor of hip or knee osteonecrosis? Results of 49 patients and review of the literature. Diagn. Interv. Radiol..

[21] Arriagada M., Arinoviche R. (1994). X-ray bone densitometry in the diagnosis and followup of reflex sympathetic dystrophy syndrome. J. Rheumatol..

[22] Cepollaro C., Gonnelli S., Pondrelli C., Martini S., Montagnani A., Rossi B., Gennari C. (1998). Usefulness of ultrasound in Sudeck’s atrophy of the foot. Calcif. Tissue Int..

[23] de Mos M., Huygen F., Dieleman J.P., Koopman J., Stricker C.B.H., Sturkenboom M. (2008). Medical history and the onset of complex regional pain syndrome (CRPS). Pain.

[24] Oehler N., Rolvien T., Schmidt T., Butscheidt S., Oheim R., Barvencik F., Mussawy H. (2019). Bone microstructure is significantly altered in CRPS-affected distal tibiae as detected by HR-pQCT: A retrospective cross-sectional study. J. Bone Miner. Metab..

[25] Bouvier M., Colson F., Noel E., Tebib J.G., Felman C. (1997). Two new case-reports of reflex sympathetic dystrophy syndrome in patients with osteogenesis imperfecta. Review of the literature. Rev. Rhum. Engl. Ed..

[26] Varenna M., Crotti C. (2018). Bisphosphonates in the treatment of complex regional pain syndrome: Is bone the main player at early stage of the disease?. Rheumatol. Int..

[27] Varenna M., Orsini F., Di Taranto R., Zucchi F., Adami G., Gatti D., Crotti C. (2024). Bone Turnover Markers and Wnt Signaling Modulators in Early Complex Regional Pain Syndrome. A Pre-specified Observational Study. Calcif. Tissue Int..

[28] Oehler N., Mussawy H., Schmidt T., Rolvien T., Barvencik F. (2018). Identification of vitamin D and other bone metabolism parameters as risk factors for primary bone marrow oedema syndrome. BMC Musculoskelet. Disord..

[29] Lee S.U., Na K.T., Lee Y.M., Park J.H., Joo S.Y. (2020). Low vitamin D levels in post-menopausal women are associated with complex regional pain syndrome type I in surgically treated distal radius fractures. J. Orthop. Surg. Res..

[30] Eidmann A., Eisert M., Rudert M., Stratos I. (2022). Influence of Vitamin D and C on Bone Marrow Edema Syndrome—A Scoping Review of the Literature. J. Clin. Med..

[31] Klontzas M.E., Vassalou E.E., Zibis A.H., Bintoudi A.S., Karantanas A.H. (2015). MR imaging of transient osteoporosis of the hip: An update on 155 hip joints. Eur. J. Radiol..

[32] Asadipooya K., Graves L., Greene L.W. (2017). Transient osteoporosis of the hip: Review of the literature. Osteoporos. Int..

[33] Krämer H.H., Hofbauer L.C., Szalay G., Breimhorst M., Eberle T., Zieschang K., Rauner M., Schlereth T., Schreckenberger M., Birklein F. (2014). Osteoprotegerin: A new biomarker for impaired bone metabolism in complex regional pain syndrome?. Pain.

